# Application of Artificial Teeth by the Auroplastic Principle

**Published:** 1854-01

**Authors:** A. Hill


### ARTICLE III.

### *Application of Artificial Teeth by the Auroplastic Principle.*

NORWALK, CT., *Nov. 9th*, 1853.

DR. C. A. HARRIS:

DEAR SIR—In the October No. of the American Journal, I noticed an article entitled, "Application of Artificial Teeth, by the Auroplastic Principle, by Edwin Truman, Dentist, London."

In referring to this article in your editorial, you observe, "In alluding to the use of gutta-percha as a base for artificial teeth in a former number of the Journal, we stated that we believed Dr. Hill, of Norwich, (should be Norwalk, Ct.,) was the first to test its applicability for this purpose. He informed us by letter, about four years ago, if our memory serves us correctly, that he had then made some experiments with it, which promised the most satisfactory results. We should be glad to hear from him again upon the subject, and learn to what extent his expectations had, by his subsequent experience, been realized."

It will be my pleasure to respond to the wish so courteously expressed in the paragraph just quoted, and I will now proceed to give the result of some experiments with the substance referred to.

My experiments with *pure* gutta-percha as a base for artificial teeth, were by no means satisfactory. It is too stringy and elastic in its pure state, to be used with much convenience. I therefore made a compound, similar to the article known as "Hill's Stopping"—with which I was much better satisfied, and which I subsequently used as a base for artificial teeth.

With this compound material, my experiments have been chiefly made. My method is as follows: After having secured, and prepared my plaster model, I cut out a rough pattern for a plate, from the compound material, which is rolled out into a sheet about the sixteenth of an inch in thickness. This, I immerse in hot water, when it immediatly becomes soft and plastic. While plastic, I press it with my thumb and fingers where I wish it to go upon the model. Occasionally immersing it in the hot water as it becomes cool, until it is made to take the precise shape, which I desire. It is now to be trimmed, and dressed until the edges are smooth and true.

It is necessary that the alveolar border should be quite thick. This can be easily made so, by adding small strips, until the required thickness is obtained. When this is done, it is ready to receive the teeth, which may be secured as follows:

If pivot teeth are used, (and they can be in many cases with satisfaction,) I proceed as follows: I sit down, with my model before me, with a small spirit lamp lighted at my right hand. After selecting the tooth which I design to use, I seize it with a pair of common pliers and gradually heat it in the blaze of the spirit-lamp, (it requires but little heat,) and then press it firmly in its bed on the alveolar border, just as I wish it to stand when in the mouth. And thus I proceed, until the teeth are arranged, occasionally trying the piece in the mouth, to see that it is all right. Supposing the teeth to be all right in their arrangement, I next proceed with a small flat-smooth pointed instrument to lay the compound all around the base of the teeth, in a solid and substantial manner, occasionally heating my instrument in the blaze of the spirit-lamp, and massing it down.

If ordinary plate teeth are used, they should be lined, or backed in the usual manner for setting on plate, only leaving the backings longer than is customary for soldering on plate, and allowing it to turn out a little from the tooth, so that the compound can cover it, and retain it firmly in its place. This can be done so as to give great strength to the teeth, when properly arranged. All this can be done in less time than it will require to write these directions, save, perhaps, the backing of the teeth.

I suppose the teeth now in use in Dr. Allen's process of continuous gums will answer a better purpose for this kind of work. Such, in short, is my plan of setting teeth, on a compound base. It will be perceived, that any required shape can be given to the gums, and any irregularity in the alveolar ridge easily adjusted. A portion of the compound I prepare of a gum color, and lay it on as may suit my convenience or taste, with my small smooth-flat instrument, heated in the blaze of my spirit lamp. The whole process is very simple, and much easier executed than the method adopted by Mr. Truman of London.

Let us consider the objections to this way of mounting artificial teeth. The objection does not lie on the ground of nonretention of the teeth. If proper care is observed in mounting them, they will bear any legitimate use in mastication. They will also continue useful for several years, as my experiments will show.

But the great objection in my mind is, they will not retain their proper color. I have succeeded in making a beautiful gum colored compound, and can paint them with various shades of the same article, and when first used, look exceedingly well, but they will change color in the mouth, and look bad enough after a little use. If this difficulty could be obviated, I should think much better of the plan. But I see not how it can be, until we can impart to it an enameled surface, which is not likely soon to be accomplished. A flesh colored compound, would indeed be desirable, but I have not yet succeeded in making a gum color that would not fade, or turn dark and livid, under the influence of the action of the fluids of the mouth. A simple compound like the "stopping," operates the best in the long run, of any I have tried.

Temporary sets may be constructed on this plan, with great facility, and at a small expense, answering a very good purpose. Indeed, I should think them altogether preferable to the English bone, or hippopotamus base. And they may be renewed or entirely reconstructed at any time, with little difficulty.

For under sets, this article is peculiarly adapted, inasmuch as it is so easily moulded to any desirable form. And the base can be made so thick and strong, as to endure any service. And the teeth are firmly held in their places by being stuck into the compound when slightly heated, without any other support. It will afford sufficient weight to the piece, and bulk, to any extent. Where one or more grinders are lost, this plan furnishes a simple and economical substitute, as they may be retained without clasps or ligatures.

If thrown back twenty years, I should certainly regard this as a great and useful invention. And even now, it may be regarded as a great convenience for temporary uses. But otherwise, I have little satisfaction in this kind of work, since I have seen the beautiful block and continuous gum work of the present day. As to strength, there is no difficulty; and in some cases it may be made really handsome. But it is not easy to overcome our predilection in favor of the precious metals, as a base for artificial teeth.

I have thus, my dear doctor, endeavored to give you a very brief description of my experiments on the "auroplastic principle," and would be pleased to communicate more specific information regarding it, either to yourself, or any other member of the dental profession, if desired so to do, at any time.

Very truly, yours,

A. HILL.

P. S. I do not understand how gold can be deposited directly on gutta-percha by means of the galvanic battery, as set forth by Mr. Truman, of London. If this can be done, a great point will be gained, as to the success of these experiments, and the principal objections obviated.

Perhaps you can give me some light upon this subject. If so, I would esteem it a special favor.

